# Retraction: Bridging the gaps in eating disorder care: a systematic and comparative review of guidelines for prevention, early intervention, and service delivery

**DOI:** 10.3389/fpsyg.2026.1794398

**Published:** 2026-02-04

**Authors:** Frontiers Editorial Office

The journal retracts the October 8 2025 article cited above.

Following concerns regarding the originality of the article, an investigation was conducted in accordance with Frontiers' policies. It was found that the complaint was valid and that the article should be retracted, because of an unacceptable level of similarity to other articles, most notably Dann KM, Harrison A, Veldre A, Hay P, Touyz S. Embracing a different outlook: Strengths and goals of individuals currently in treatment for anorexia nervosa. Eat Weight Disord. 2024 Oct 2;29(1):63. doi: 10.1007/s40519-024-01689-x.

This retraction was approved by the Chief Editors of Psychology and the Chief Executive Editor of Frontiers. The authors agree to this retraction.

